# Exploring the relationship between the density of the iris color and bipolar disorder: a case-control study, Egypt

**DOI:** 10.1186/s12991-025-00562-0

**Published:** 2025-04-28

**Authors:** Usama Mahmoud Youssef, Yasser Mohamed Raya, Mohammad Gamal Sehlo, Osama Mohamed Gado, Fayza Mohammed Hussien, Ahmed AM Gad, Mervat Said

**Affiliations:** 1 Psychiatry Department, Faculty of Medicine, Zagazig University, P. O. Box 44519, Zagazig, Egypt; 2 Ophthalmology Department, Zagazig University, Zagazig, Egypt

**Keywords:** Iris color, Eye, Bipolar disorder (BD), Mood disorders

## Abstract

**Background:**

The eyes serve as a portal to the brain and are highly connected neurologically, making them the only externally visible part of the brain. Moreover, the correlation between the physical attributes of the eye and psychiatric disorders has been increasingly established in recent years. Therefore, this study examined the association between iris color density and bipolar I disorder (BD).

**Methods:**

In a case-control study, 48 subjects diagnosed with BD are compared to an equal number of healthy controls. A semi-structured interview questionnaire is designed to collect sociodemographic, psychiatric, medical history, and clinical data from all participants. The Group with BD is diagnosed based on clinical assessment by the Consultant/specialist by using a semi-structured clinical interview for DSM 5 Clinician Version (SCID-5-CV) to diagnose BD’s current episode and to exclude the other comorbid mental disorders. Additionally, the group with BD is further assessed by The Young Mania Rating Scale (YMRS) and Hamilton Depression Rating Scale (HDRS) to evaluate the severity of manic and depressive symptoms. The iris color of participants in both groups was evaluated using a standardized photographic system for iris imaging.

**Results:**

It showed a statistically significant increase in the percentage of the colored iris in the patients’ group compared with the control group, and the presence of a colored iris significantly increases the risk of BD by 2.36 folds. There is no statistically significant difference between iris color and either hospitalization, suicide, Electroconvulsive Therapy (ECT), family history, medical history, duration, or frequency of episodes.

**Conclusion:**

Our findings revealed a strong association between iris color and bipolar disorder (BD) but no significant association between iris color and clinical factors such as hospitalization, suicide, electroconvulsive therapy (ECT), family history, medical history, or the duration and frequency of episodes. These results suggest that iris color may serve as a trait marker rather than a state marker in BD, potentially offering a simple and non-invasive indicator of bipolarity.

## Introduction

Characterized by mood disturbances, bipolar disorder (BD) is a complex and multifaceted mental illness regarded as one of the most severe mental disorders worldwide. The adult population’s estimated lifetime prevalence of BD falls within 1–3% [1]. Additionally, it is the 6th major cause of disability globally, it has been recognized by the World Health Organization (WHO) as BD [2].

Economic-wise, in the USA, the estimated direct expenses related to bipolar disorder amounted to US$30.7 billion in 2009. The majority of this figure is linked to inpatient care, with the overall rate of hospital admission ranging from 13 to 39% among those with bipolar illness. Furthermore, the direct costs encompass expenses such as outpatient medical care and non-treatment-related costs (e.g., involvement with the criminal justice system) [3]. In Egypt, bipolar disorder is a significant mental health concern, though epidemiological data remain limited. A study by Okasha et al. (2012) [4] estimated the lifetime prevalence of bipolar spectrum disorders in Egypt to be approximately 1.5%, aligning with global estimates. The study highlighted the substantial burden of BD on individuals and families, with many patients experiencing significant functional impairment and reduced quality of life. Furthermore, El Missiry et al. (2017) [5] reported that BD is associated with high rates of disability, particularly in areas such as occupational functioning, social relationships, and self-care. These findings underscore the need for early detection and effective management of BD.

It is well-reported that numerous biological, genetic, and psychosocial factors have been extensively investigated as potential contributors to the development of bipolar disorder. However, despite these efforts, no definitive mechanism for the pathogenesis of the disease has been identified [6]. Several studies have suggested that immune modulators may contribute to mood disturbances and could be accountable for symptoms [6]. Additionally, past research collectively supports the notion that alterations in cytokines disrupt homeostatic mechanisms, the immune system, neurotransmitters, and the endocrine system, ultimately leading to neuronal degeneration and impairing neurogenesis in individuals with bipolar disorder [7]. Furthermore, studies involving imaging have revealed a reduction in the white matter among individuals with bipolar disorder [8, 9], particularly in adolescent cases [10]. These structural changes highlight the progressive nature of the disorder and underscore the importance of early detection and timely intervention to mitigate its long-term impact on brain function and overall quality of life.

The eyes are considered a means of observing the brain and are so closely connected neurologically that they could be deemed the only part of the brain visible from the outside. They appear to contain essential insights into our brain’s functionality. Additionally, several genes are implicated in both eye development and normal neuronal growth [11, 12], including Semaphorin 3 A (SEMA3A) [13]. Furthermore, these genes are also associated with psychiatric disorders [13, 14].

The correlation between the physical attributes of the eye and psychiatric disorders has been increasingly established in recent years [15], particularly the linkage between schizophrenia and associated specific retinal structures [16, 17]. The human iris is the most distinctive part of the eye, and it is marked by crypts (indentations in the iris stroma), pigmented spots, and wrinkles [11, 18, 19]. Previous studies reported a relationship between iris characteristics and schizophrenia. For instance, Kent et al. (1956) [20] reported that hospitalized individuals with schizophrenia exhibited a higher prevalence of mixed iris pigmentation compared to other groups.

Another example is a study by Trixler et al. (2017) [21], which found that individuals with schizophrenia exhibited a higher incidence of pigment spots, concentric furrows, and Wolfflin nodules in their iris structure compared to healthy controls. While research directly examining iris density and mood disorders is limited, some research provides indirect evidence of a connection. For instance, Goel et al. (2002) [22] found that individuals with lighter iris colors exhibited less severe depressive symptoms in seasonal affective disorder (SAD), a subtype of depression. Additionally, Terman and Terman (2000) [23] demonstrated that lighter iris colors are associated with greater light sensitivity, which may influence mood regulation. Furthermore, genetic studies have identified shared pathways between iris development and neurobiological processes implicated in mood disorders [24]. Although these findings are preliminary and need more investigations, they suggest that iris color may indicate a possible linkage between the iris color and mood disorders.”

Several evidence-based hypotheses may explain the potential link between iris pigmentation and psychiatric conditions. First, neurodevelopmental anomalies, such as those seen in neurofibromatosis type 1 (e.g., Lisch nodules), suggest shared developmental pathways between the iris and the brain that could contribute to psychiatric disorders [25]. Second, melanin biosynthesis shares precursors with neurotransmitters like dopamine and serotonin, and disruptions in melanin production may influence mood regulation, potentially contributing to affective disorders [26]. Third, specific iris characteristics, such as pigment spots and crypts, have been correlated with schizophrenia, indicating a potential link between iris morphology and psychotic disorders [27]. Finally, neurotransmitters like serotonin regulate melanogenesis, and dysregulated neurotransmitter levels in psychiatric disorders may alter melanin synthesis and iris pigmentation [28]. These perspectives provide a comprehensive theoretical foundation for exploring iris pigmentation as a potential biomarker in psychiatric research.

To the best of our knowledge, no prior studies have investigated the relationship between iris color and bipolar disorder. Thus, this study represents the first to explore the association between iris color and BD.

## Materials and methods

### Subjects

A sample of 48 subjects (group I) with BD diagnosed according to DSM-5 Diagnostic criteria were interviewed using the Structured Clinical Interview for DSM-5 disorders, clinician version (SCID-5-CV) [29] for confirmation of the diagnosis of bipolar disorder. The patients were recruited from the inpatient ward and the outpatient clinic of the psychiatry department of Zagazig University Hospital (Z.U.H.), Egypt, within 12 months, from November 2020 to November 2021. This sample was recruited by using a systematic random sampling technique. The patients were compared with 48 healthy controls (group II) matched with the patients for age, gender, and education enrolled from nurses, workers, and doctors working at Z.U.H. and also from the medical students of Zagazig University.

Eligibility for participation was determined based on predefined inclusion and exclusion criteria. Patients diagnosed with BD according to DSM-5 criteria and aged 18 to 60 years were enrolled in the study. For the control group, healthy individuals matched to patients by age, gender, and educational level were included.

### The exclusion criteria

For the Patients ‘group, subjects with comorbid psychiatric diagnoses or major medical illnesses that could affect psychological conditions (such as cancer or autoimmune disorders) and substance abuse were not included. Exclusion criteria were assessed through detailed history-taking and a thorough review of the patient’s medical records. The exclusion criteria for the controls are the presence of any psychiatric illness, major medical illness, or substance abuse. The study obtained ethics approval from the Zagazig University Institutional Review Board (IRB) (NO: ZU-IRB #5911/10-2-2020), and all procedures adhered to the ethical principles stated in the Declaration of Helsinki and its subsequent revisions. Additionally, all participants provided written consent before taking part in the study.

### Research design

This is a case-control study, 48 patients diagnosed with bipolar I disorder (BD) are compared to an equal number of healthy controls to assess the frequency of low-density iris color and to analyze its relation to bipolar disorder. The sample size was determined using Open Epi software, with a study power of 80% and a confidence level of 95%. Based on a previously published study [30] conducted among mood disorder patients, which reported a prevalence of low-density iris color at 36% compared to 16% for high-density iris color, the estimated sample size required for this study was calculated to be 48 patients diagnosed with bipolar disorder and 48 healthy controls. This calculation ensures adequate statistical power to detect significant differences between the groups. Socio-demographic data (age, sex, marital state, education, occupation, residence) and full psychiatric and medical histories were taken for all the participants.

Assessment of psychopathology: The BD group is confirmed through a clinical assessment conducted by either a consultant or two specialists. This process involves a semi-structured clinical interview based on the DSM-5 Clinician Version (SCID-5-CV) to confirm the diagnosis of BD and rule out other comorbid mental conditions [31]. The validity and reliability of this assessment tool are supported by previous studies [32]. To assess the severity of the bipolar episode, the Young Mania Rating Scale (YMRS) is employed for manic episodes, and the Hamilton Depression Rating Scale (HDRS) is utilized for depressive episodes. The YMRS is widely recognized in clinical trials as the primary standardized instrument for evaluating manic symptoms in bipolar disorder. According to Young et al. (1978) [33], the scale demonstrates satisfactory inter-rater reliability, with a total score reliability of 0.93 and item reliability ranging from 0.67 to 0.95. The YMRS consists of 11 items, scored by an interviewer, with four items rated on a scale of 0 to 8 (irritability, speech, thought content, and disruptive/aggressive behavior) and the remaining seven rated on a scale of 0 to 4. Scores range from 0 to 60, with averages of 13 indicating minimal severity, 20 for mild, 26 for moderate, and 38 for severe episodes.

On the other hand, the Hamilton depression rating scale is a prevalent tool for evaluating depression administered by clinicians. It is intended for use in adults and aims to measure the intensity of their depression by exploring their mood, guilt feelings, thoughts of suicide, sleeping problems, restlessness or slowness, nervousness, loss of weight, and physical symptoms. The HDRS17 consists of 17 elements, and each element is evaluated on a scale of either 3 or 5 points, depending on the symptom. The questions pertain to signs of depression observed during the previous week. A score of 0 to 7 is considered typical or shows clinical remission, while a rating of 20 or above indicates a moderately severe condition [34]. The Arabic version is validated by Al Rabeeh et al. (2007) [35]. Additionally, a simple questionnaire was designed to assess the clinical characteristics of BD in the patients’ group, including the following: age of onset of the disorder, duration of the disorder, frequency of manic/ depressive episodes, number of hospitalizations, history of suicide (thoughts or attempts), family history of psychiatric illness (including bipolarity).

**Fig. 1:**
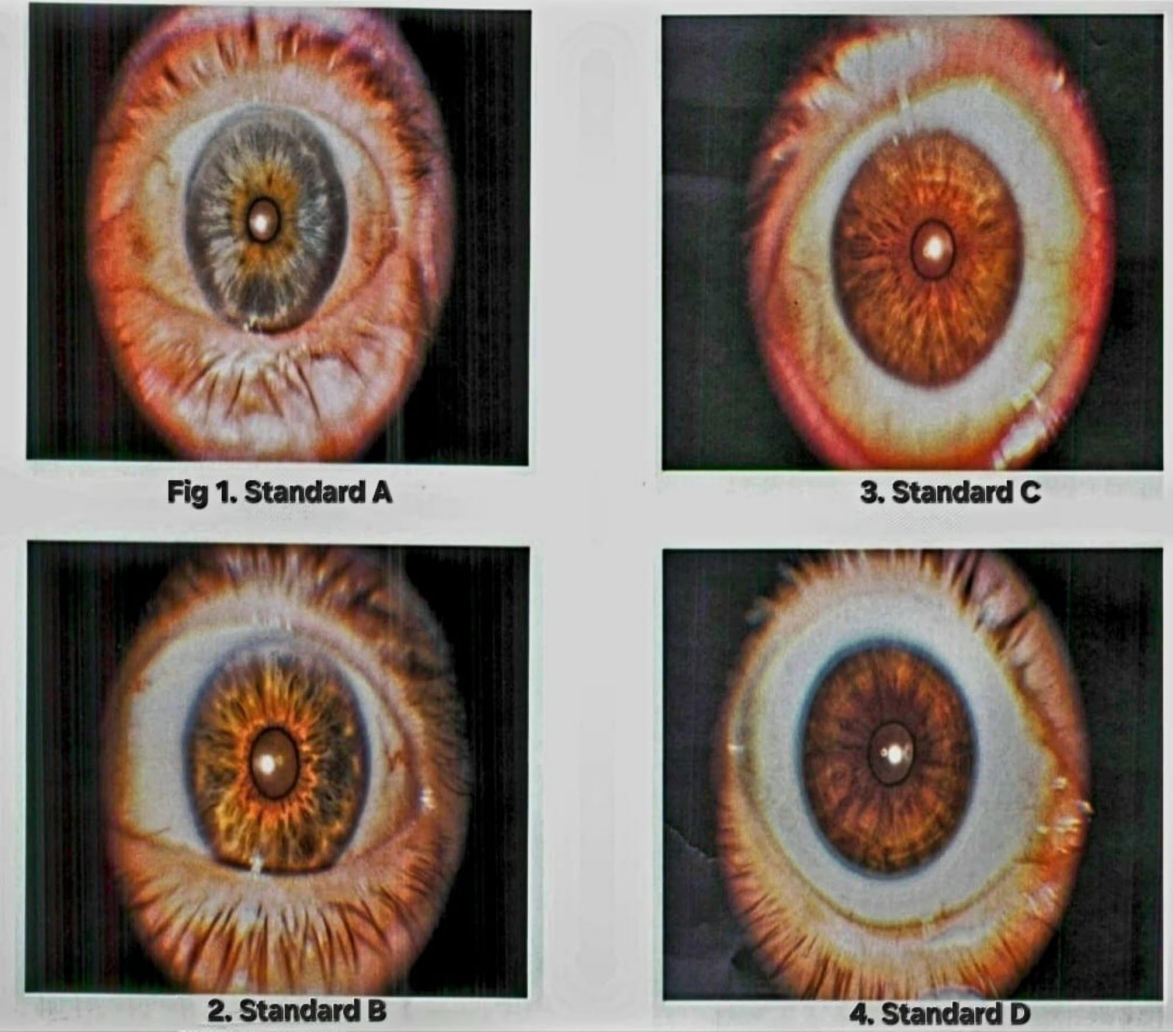
Derived from the iris’s classifications by Seddon et al. (1990) [36]

Regarding detecting iris color among the participants, in 1990, Seddon et al. [36]. created a method of categorizing iris color using standard photographs. The classification system identifies the primary color of the iris (blue, gray, green, light brown, or brown) and the quantity of brown or yellow pigment found in it. Based on these factors, various categories of iris color are established. They utilized a grading system with five grades, which are as follows:

- Grade 1: This refers to a blue or gray iris with brown or yellow specks that are equivalent to or less than the percentage found in standard A (Fig. 1).
- Grade 2: This includes a blue, gray, or green iris with brown or yellow specks that are more significant than those found in standard A but equal to or less than those found in standard B (as shown in Fig. 2).
- Grade 3: This pertains to a green or light brown iris with brown or yellow specks that are greater than those found in standard B but equal to or less than those found in standard C (as shown in Fig. 3).
- Grade 4: This refers to a brown iris with a color that is equal to or less than that found in standard D (Fig. 4) but greater than those found in standard C.
- Grade 5: This is a darker brown iris than the one found in standard D.

All participants, including both case and control groups, underwent iris imaging using a high-quality digital camera under standardized lighting conditions and camera settings. A ring-shaped lamp was used to ensure consistent eye illumination. Iris’s color was classified according to Seddon’s system based on the captured images. For more simplifications and seeking statistical analysis, participants who are identified as grades 1 to 4 are grouped as the low-density iris categories and grade 5 as the high-density category in both groups (cases and control groups). The assessment of iris color density for all participants was conducted under the supervision of the ophthalmologist, one of the co-authors.

### Statistical analysis

Data analysis was conducted using version 26 of the SPSS (Statistical Package for the Social Sciences) software. The size of the sample was determined using the open epi software. Descriptions of quantitative variables included their means and standard deviations, or median and range, based on the type of data. Categorical variables were described using absolute frequencies and compared using the chi-square test. To compare ordinal data between the two groups, the chi-square for the trend test was used. The Kolmogorov-Smirnov test was used to verify the distribution type, while the Levene test was used to verify the homogeneity of variances, both to ensure the use of parametric tests. The Mann-Whitney test was used for not normally distributed data, and the independent sample t-test for normally distributed data was used to compare quantitative data between two groups. To compare continuous quantitative variables between more than two groups, the one-way ANOVA test was used. The ROC curve was used to determine the best cutoff of a certain quantitative parameter in the diagnosis of certain health problems. The level of statistical significance was set at *P* < 0.05. A highly significant difference was present if *p* ≤ 0.001.

## Results

### Demographic and general clinical data of enrolled patients

On comparing the patient group (Group I) with the control group (Group II) according to the sociodemographic data. There was a statistically non-significant difference between the two studied groups regarding age, sex, marital status, education, occupation, or residence (Table 1).

**Table 1:** Comparison between the studied groups regarding demographic data:

Parameter	Patient group	Control group	χ^2^/t	*P*
	*n* = 48 (%)	*n* = 48 (%)		
**Gender**:			0.664	0.415
Female Male	24 (50%) 24 (50%)	20 (41.7%) 28 (58.3%)		
**Age (year)**:				
Mean ± SD	36.35 ± 11.76	33.9 ± 9.8	1.113	0.269
**Marital status**:				
Divorced Married Single Widow	2 (4.2%) 29 (60.4%) 13 (27.1%) 4 (8.3%)	0 (0%) 31 (64.6%) 15 (31.3%) 2 (4.2%)	MC	0.594
**Occupation**:				
No/housewife/student Worker Free work/merchant Professional	28(58.3%) 5 (10.4%) 11(22.9%) 4(8.3%)	17(35.3%) 14(29.2%) 14(29.2%) 3(6.3%)	1.46^§^	0.227
**Education**:				
Illiterate Preparatory Secondary High	4 (8.3%) 7 (14.6%) 30 (4.2%) 7 (14.6%)	2 (4.2%) 10 (20.8%) 28 (58.3%) 8 (716.7%)	0.073^§^	0.787
**Residence**:				
Rural Urban	15 (31.3%) 33 (68.7%)	22 (45.8%) 26 (54.2%)	2.155	0.142

χ^2^Chi square test. ^§^chi square for trend test t independent sample t-test MC Monte Carlo test

-Assessing the clinical characteristics of the patient group (Group I), Thirty patients (62.5%) had a previous history of hospitalization, six patients (12.5%) had a history of suicidal attempts/ideation, 52.1% received ECT, 52.1% had a positive family history of psychiatric illness, and 50% were compliant with treatment. Duration of episodes The Duration of illness ranged from 1 month to 55 years, with a mean of 26.33 years. The frequency of episodes ranged from 1 to 28, with a median of 4 and mean ± SD of 6.5 ± 5.2. Hamilton’s score ranged from 13 to 35 with a mean of 26.33, while Mania’s score ranged from 21 to 39 with a mean of 28.77. (Table 2).

### Comparing the two groups regarding iris color

There is a statistically significant increase in the percentage of low-density iris in the patients’ group (66.7%) compared with the control group (45.8%), and the presence of a low-density iris significantly increases the risk of bipolar disorder by 2.36 folds (confidence interval 1.03–5.4). The group of low-density iris included subjects under categories 1,2,3 and 4, and a group of high-density iris included category 5 based on classification by Seddon et al. 1990. (Table 3).

- There is a statistically non-significant relation between the presence of euthymia, depressive or manic episodes, and iris color. Patients with low-density iris represented 68.8%, 66.7%, and 64.7% of those with euthymia, depressive and manic episodes, respectively. (Table 4).
- There is a statistically non-significant relation between iris density and either hospitalization, history of suicide, ECT administration, family history of psychiatric disorder, duration of illness, or frequency of episodes. (Table 5).

**Table 2:** Distribution of the studied patients according to some specific clinical data

	*n* = 48	%
**Number of hospitalizations:**		
No Yes	18 30	37.5% 62.5%
**History of Suicide**:		
No Yes	42 6	87.5% 12.5%
**ECT administration**:		
No Yes	23 25	47.9% 52.1%
**Family history**:		
Negative Positive	23 25	47.9% 52.1%
**Compliance with treatment**:		
No Yes	24 24	50% 50%
	**Median**	**Range**
**Duration of illness**	**12 years**	**1 month – 55years**
	**Mean ± SD**	**Range**
**Hamilton scale mean score (** ***n*** ** = 15)**	26.33 ± 6.23	13–35
**YMRS mean score (** ***n*** ** = 17)**	28.77 ± 5.21	21–39
**Frequency of episodes**	6.5 ± 5.2.	1–28

YMRS: Young mania rating scale

**Table 3:** Comparison between the studied groups regarding iris density

Parameter	Case group	Control group	χ^2^	*P*
	*n* = 48 (%)	*n* = 48 (%)		
**Low density Iris** (Category 1–4) **High density Iris** (Category 5)	32 (66.7%) 16 (33.3%)	22 (45.8%) 26 (54.2%)	18.5873	0.04*
**COR (95% CI)**		2.36 (1.03–5.4)		

χ^2^Chi square test **p ≤ 0.001 is statistically highly significant COR Crude odds ratio CI confidence interval

**Table 4:** Relation between different staging of bipolar patients and iris density:

Parameter	Euthymia	Depressive	Manic	χ^2^	*P*
	***n*** ** = 16(%)**	***n*** ** = 15(%)**	***n*** ** = 17(%)**		
**Low density Iris** **High density Iris**	11 (68.8%) 5 (31.2%)	10 (66.7%) 5 (33.3%)	11 (64.7%) 6 (35.3%)	0.061	0.97

χ^2^Chi square test

**Table 5:** Relation between iris density and clinical data of studied patients

Parameter	Low density Iris	High density Iris	χ^2^	*P*
	*n* = 32 (%)	*n* = 16 (%)		
**Number of hospitalizations:**				
No Yes	12 (37.5%) 20 (62.5%)	6 (37.5%) 10 (62.5%)	0	> 0.999
**History of Suicide**:				
No	26 (81.3%)	16 (100%)	Fisher	0.064
Yes	6 (18.7%)	0 (0%)		
**ECT administration**: No Yes	13 (40.6%) 19 (59.4%)	10 (62.5%) 6 (37.5%)	2.045	0.153
**Family history of psychiatric illness**:				
Negative Positive	13 (40.6%) 19 (59.4%)	10 (62.5%) 6 (37.5%)	2.045	0.153
**Compliance with treatment**: No Yes	13 (40.6%) 19 (59.4%)	11 (68.8%) 5 (31.3%)	3.375	0.066
	**Median(IQR)**	**Median(IQR)**	**Z**	**P**
*Duration (year)	11.5 (4–19.5)	15 (1.25–22.25)	-0.449	0.653
**Frequency	4 (2–10)	8 (2.25–10)	-0.891	0.373

χ^2^Chi square test Z Mann Whitney test IQR interquartile range *Duration: Total time (in years) since the onset of bipolar disorder. **Frequency: Total number of manic and depressive episodes experienced by the patient

## Discussion

To the best of our knowledge, no prior studies have investigated the association between iris color and bipolar disorder. This study represents one of the pioneering studies to evaluate this relationship on a global scale.

The most striking finding in this study is the statistically significant increase in the percentage of the low-density iris in the patients’ group (66.7%) compared with the control group (45.8%), and the presence of a low-density iris significantly increases the risk of BD by 2.36 folds (confidence interval 1.03–5.4). This finding is consistent with Chandola’s (2016) [30] study, which included 300 participants of both sexes. Eye color data were collected for all participants as part of the observational analysis. The study reported that males with light brown eye color had significantly higher psychiatric morbidity (68.47%) compared to females, with the difference being statistically significant (*p* < 0.01). However, a study by Goel et al. (2002) [22] found that blue-eyed (low-density iris) patients with seasonal affective disorder (SAD) exhibited significantly lower levels of depression and fatigue compared to darker-eyed (high-density iris) patients. These findings may provide context for interpreting our results.

An explanation of why low-density iris patients with SAD are less likely to be depressed or fatigued during winter (in the previous studies) may help in thinking about the relationship between BD and the low-density iris (in our study). The hypothesis suggests that the absence of pigmentation in the eyes of blue-eyed individuals reduces their susceptibility to the reduced natural light during the winter, as it is commonly known that they are more sensitive to light. Research conducted by Terman and Terman in 1999 [23] found that blue-eyed patients displayed greater enhancements in cone sensitivity during the summer months compared to those with darker eyes. Building on our findings, further studies with larger patient samples are needed to confirm this relationship. Such research may pave the way for discovering new genetic links associated with BD in individuals with low-density iris pigmentation across diverse demographic and geographic settings on a global scale.

According to a study by Steven and Silverstein (2015) [37], schizophrenia is associated with several eye-related structural and physiological disturbances that may contribute to impaired visual processing and altered visual experiences in affected individuals. These disturbances include widened retinal venules (potentially due to tissue loss), loss of ganglion cell axons and possibly cell bodies, abnormalities in photoreceptor dopaminergic activity, decreased lateral inhibition related to GABA and glycine, various retinopathies and maculopathies, cataracts, and poor visual acuity. The variability in these eye-related phenomena has been shown to impact performance on visual processing tasks that schizophrenia patients typically struggle with. Furthermore, the study proposes that aspects of retinal structure and function can be utilized as biomarkers for brain pathology and disease progression in schizophrenia.

In 2013, Bittencourt et al. [38] conducted a study to examine the connection between oculomotor performance and psychopathology, including depression, BD, schizophrenia, attention-deficit hyperactivity disorder, and anxiety disorder. The study indicated that changes in saccadic eye movements are strongly linked to psychiatric disorders and could potentially serve as a marker for certain conditions. In 2022, Tian et al. [39] conducted another study that showed a higher frequency of pigment spots and iris crypts in individuals with schizophrenia compared to healthy individuals. The results of the logistic regression analysis suggested that the total number of iris crypts and pigment spots was linked to an increased risk of schizophrenia. Furthermore, Fernandes (2017) [40] examined color vision in patients with BD and observed lower color discrimination abilities in patients compared to controls. Our results showed that there is no statistically significant difference between iris color and different staging of bipolar disorder, number of hospitalizations, history of suicide, ECT administration, family history of psychiatric illness, duration, or frequency of episodes. These findings suggest that iris color may serve as a trait marker rather than a state marker in individuals with bipolar disorder.

Our study also has some limitations. Firstly, it is a cross-sectional study that confirms an association between the low-density iris and BD but not causality. Secondly, the small sample size limited the generalizability of our study and restricted the depth of statistical analysis, such as the ability to perform multivariate analysis. Therefore, we recommend that future studies address this limitation by including larger sample sizes. However, our study has a major strength in that this is one of the leading studies that evaluate the relationship between iris color and BD in Egypt, so it opens the floor for further studies to confirm our results and for longitudinal studies that prove causality.

## Conclusion

In summary, our study revealed that the presence of a low-density iris was associated with a 2.36-fold increased risk of bipolar disorder, suggesting a potential link between iris characteristics and the disorder. However, this association does not imply causation and future studies are needed to explore the underlying biological mechanisms. However, there was no statistically significant relationship between iris color density and different stages of BD, number of hospitalizations, history of suicide, ECT administration, family history of psychiatric illness, duration of illness, and frequency of episodes. These findings suggest that iris color may be considered a trait marker in bipolar patients, detectable in a simple manner, which could provide insights into the biological underpinnings of bipolar disorder. However, further research is needed to confirm whether this association reflects a causal relationship, shared biological pathways, or a specific genetic linkage.

## Data Availability

No datasets were generated or analysed during the current study.

## Abbreviations

BD: Bipolar I disorder (BD)
SCID-5-CV: Semi-structured clinical interview for DSM 5 Clinician Version
YMRS: Young Mania Rating Scale
HDRS: Hamilton Depression Rating Scale
ECT: Electroconvulsive Therapy
WHO: World Health Organization
Z.U.H.: Zagazig University Hospital
IRB: Institutional Review Board
SPSS: Statistical Package for the Social Sciences
SAD: Seasonal affective disorder
